# Administering a subconjunctival injection

**Published:** 2009-03

**Authors:** Sue Stevens

**Affiliations:** Nurse Advisor, Community Eye Health Journal, International Centre for Eye Health, London School of Hygiene and Tropical Medicine, Keppel Street, London WC1E 7HT, UK.

**Figure:**
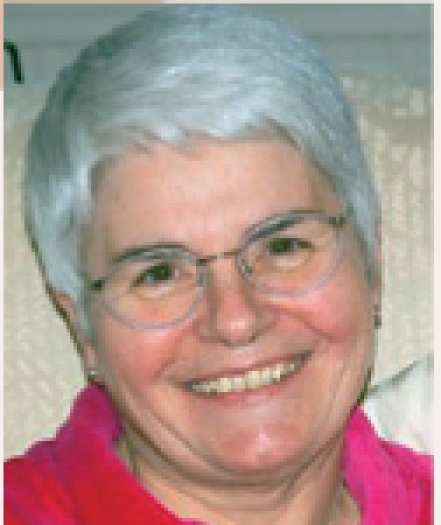


## Indications

To administer medication in high concentration:

- for severe inflammation
- for treating infection
- at the end of an operation
- for dilating the pupil.

## You will need

- local anaesthetic drops
- sterile 2 ml syringe
- prescribed medication
- sterile 21G needle
- sterile 25G needle
- eye pad
- tape
- bandage
- clean cotton wool or gauze swabs.

## Preparation

**Remember!**

**This procedure can be very distressing for the patient and needs to be explained sensitively to ensure maximum cooperation.**

- Position the patient lying comfortably with his/her head supported on a pillow.
- Reassure the patient that adequate anaesthetic drops will be instilled before the injection is given.
- Instill anaesthetic drops at five-minute intervals over half an hour - a minimum of six drops.
- Away from the patient's view, draw up the prescribed medication using the 21G needle (Figure 1).
  **Figure 1:**
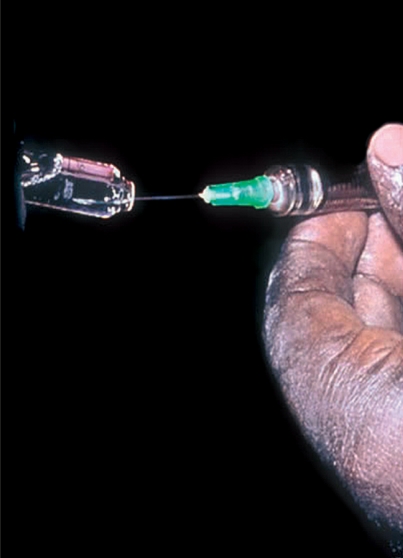
Murray McGavin
- Change to the 25G needle (Figure 2).
  **Figure 2:**
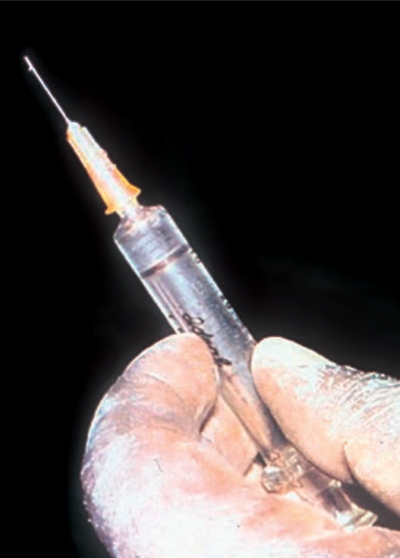
Murray McGavin

## Method

- Choose the site for the injection in the lower or upper fornix and, as appropriate, raise or lower the eyelid.
- Ask the patient to look in the opposite direction and fix his/her gaze.
- Tell the patient to expect a slight pressure sensation and keep reassuring him/her.
- With the bevel of the needle uppermost, lay the needle against the globe, away from the cornea, and make a ‘pocket’ of conjunctiva (Figure 3).
  **Figure 3:**
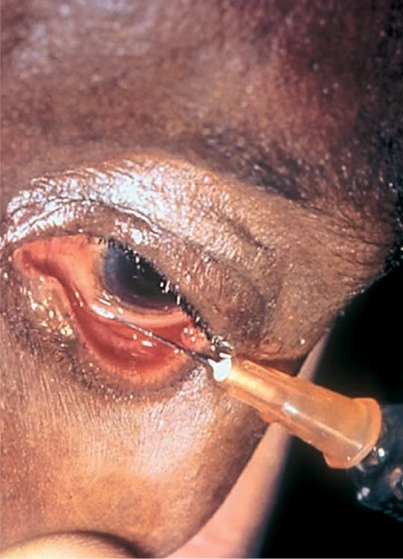
Murray McGavin
- Insert the needle into the space between the conjunctiva and the sclera.
- Ensure the bevel remains under the conjunctiva and inject the fluid slowly to create a ballooning effect (Figure 4).
  **Figure 4:**
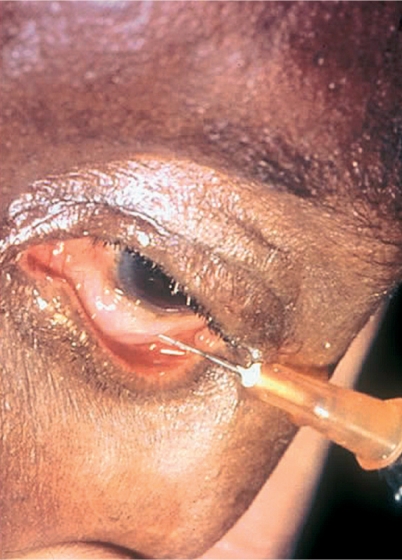
Murray McGavin
- Withdraw the needle carefully and ask the patient to close the eye - check that the eyelids can close properly.
- Dispose of the needle and syringe in an appropriate container.
- Hold an eye pad in position for a minute or so - the ballooning will subside.
- Apply the eye pad securely and, for maximum comfort, also apply a bandage.
- The pad and bandage should remain in position for two hours, after which any prescribed topical eye medication may be continued.
- Tell the patient that the conjunctiva may appear red and swollen and that the eye may be sore after the anaesthetic drops wear off. Oral paracetamol (two tablets) may be given for pain relief.

